# Multicentre evaluation of the Boehringer Mannheim/Hitachi 917 analysis system

**DOI:** 10.1155/S1463924600000080

**Published:** 2000

**Authors:** Jeroen D. E. van Suijlen, Bert G. Blijenberg, Jörg Hofmann, Kurt Bauer, Zahur Zaman, Norbert Blanckaert, Peter Degenhard, Klaus Wielckens, Carmen Ferré, Antonio Torralba, Mary Martyn, Anne Kelly, Ferrucio Ceriotti, Pierangelo A. Bonini, Wolfgang Bablok, Margaret McGovern, Wolfgang Stockmann

**Affiliations:** 1 Academisch Ziekenhuis Rotterdam Rotterdam The Netherlands; 2 SMZ-Donauspital Wien Austria; 3 Academisch Ziekenhuis Leuven Leuven Belgium; 4 Universitätskrankenhaus Köln Germany; 5 Ciutat Sanitària i Università ria de Bellvitge Barcelona Spain; 6 Royal Alexandra Hospital Paisley UK; 7 Ospedale San Raffaele Milano Italy; 8 Roche Diagnostics GmbH Mannheim Germany

## Abstract

The new selective access analysis system BM/Hitachi 917 was evaluated in an international multicentre study, mainly according to the ECCLS protocol for the evaluation of analysers in clinical chemistry. Forty-three different analytes, covering 56 different methods enzymes, substrates, electrolytes, specific proteins, drugs and urine applications were tested in seven European clinical chemistry laboratories. Additionally, the practicability of the BM/ Hitachi 917 was tested according to a standardized questionnaire. Within-run CVs (median of 3 days) for enzymes, substrates and electrolytes were <2% except for creatine-kinase MB isoform and lipase at low concentration. For proteins, drugs and urine analytes the within-run CVs were < 4% except for digoxin and albumin in urine. Between-day median CVs were generally < 3% for enzymes, substrates and electrolytes, and < 6% for proteins, drugs and urine analytes, except for lipase, creatine kinase and MB isoform, D-dimer, glycosylated haemoglobin, rheumatoid factors, digoxin, digitoxin, theophylline and albumin in urine in some materials. Linearity was found according to the test specifications or better and there were no relevant effects seen in drift and carry-over testing. The interference results clearly show that also for the BM/Hitachi 917 interference exists sometimes, as could be expected because of the chemistries applied. It is a situation that can be found in equivalent analysers as well. The accuracy is acceptable regarding a 95–105% recovery in standard reference material, with the exception of the creatinine Jaffé method. Most of the 160 method comparisons showed acceptable agreement according to our criteria: enzymes, substrates, urine analytes deviation of slope ± 5%, electrolytes ± 3%, and proteins and drugs ± 10%. The assessment of practicability for 14 groups of attributes resulted in a grading of one–three scores better for the BM/Hitachi 917 than the present laboratory situation. In conclusion, the results of the study showed good analytical performance and confirmed the usefulness of the system as a consolidated workstation in medium-sized to large clinical chemistry laboratories.


Journal of Automated Methods & Management in Chemistry, Vol. 22, No. 3 (May–June 2000) pp. 65–81

### Multicentre evaluation of the Boehringer Mannheim/Hitachi 917 analysis system

**Jeroen D. E. van Suijlen, Bert G. Blijenberg, Jörg Hofmann[1], Kurt Bauer[1], Zahur Zaman[2], Norbert Blanckaert[2], Peter Degenhard[3], Klaus Wielckens[3], Carmen Ferré[4], Antonio Torralba[4], Mary Martyn[5], Anne Kelly[5], Ferrucio Ceriotti[6], Pierangelo A. Bonini[6], Wolfgang Bablok[7], Margaret McGovern[7] and Wolfgang Stockmann[7]**

*Academisch Ziekenhuis Rotterdam, Rotterdam, The Netherlands, [1] SMZ-Donauspital, Wien, Austria, [2] Academisch Ziekenhuis Leuven, Leuven, Belgium, [3] Universitätskrankenhaus, Köln, Germany, [4] Ciutat Sanitària i Universitària de Bellvitge, Barcelona, Spain, [5] Royal Alexandra Hospital, Paisley, UK, [6] Ospedale San Raffaele, Milano, Italy, [7] Roche Diagnostics GmbH, Mannheim, Germany*

*The new selective access analysis system BM/Hitachi 917 was evaluated in an international multicentre study, mainly according to the ECCLS protocol for the evaluation of analysers in clinical chemistry. Forty-three different analytes, covering 56 different methods—enzymes, substrates, electrolytes, specific proteins, drugs and urine applications—were tested in seven European clinical chemistry laboratories. Additionally, the practicability of the BM/Hitachi 917 was tested according to a standardized questionnaire. Within-run CVs (median of 3 days) for enzymes, substrates and electrolytes were <2% except for creatine-kinase MB isoform and lipase at low concentration. For proteins, drugs and urine analytes the within-run CVs were <4% except for digoxin and albumin in urine. Between-day median CVs were generally <3% for enzymes, substrates and electrolytes, and <6% for proteins, drugs and urine analytes, except for lipase, creatine kinase and MB isoform, D-dimer, glycosylated haemoglobin, rheumatoid factors, digoxin, digitoxin, theophylline and albumin in urine in some materials. Linearity was found according to the test specifications or better and there were no relevant effects seen in drift and carry-over testing. The interference results clearly show that also for the BM/Hitachi 917 interference exists sometimes, as could be expected because of the chemistries applied. It is a situation that can be found in equivalent analysers as well. The accuracy is acceptable regarding a 95–105% recovery in standard reference material, with the exception of the creatinine Jaffé method. Most of the 160 method comparisons showed acceptable agreement according to our criteria: enzymes, substrates, urine analytes deviation of slope ±5%, electrolytes ±3%, and proteins and drugs ±10%. The assessment of practicability for 14 groups of attributes resulted in a grading of one–three scores better for the BM/Hitachi 917 than the present laboratory situation. In conclusion, the results of the study showed good analytical performance and confirmed the usefulness of the system as a consolidated workstation in medium-sized to large clinical chemistry laboratories.*

### Introduction

The Boehringer Mannheim/Hitachi 917 analysis system (BM/Hitachi 917) is the most recent medium to large-sized analysis system which was introduced to the market by Boehringer Mannheim GmbH in December 1994. The functionality under simulated routine conditions was already tested during an international field study in 11 European countries [1].

In contrast to previous analysis systems of comparable size, e.g. BM/Hitachi 717 or 737, the new 917 system offers features making it attractive to different purposes and different sections of clinical laboratories, e.g. a high number of reagent channels, convenient reagent handling, convenient calibration, flexible application settings, short-term applications and automatic predilution. BM/Hitachi 917 can either be used as a kind of 'workhorse' for the most often requested analytes or as a consolidated workstation for the determination of at least 48 different analytes on board covering, besides the classical routine assays, specific protein methods, drug methods in serum and urine, and urine applications for enzymes, substrates, electrolytes and proteins. The analyser is designed as a closed system with a special reagent line and fixed applications; however, five user-defined methods can be set by the operator. A software upgrade was introduced in April 1996; a draft version was tested at the end of the multicentre study. This software version offers more convenience for the operator and an enhanced data management system, and has built-in features related to accreditation aspects.

The versatility of the new instrument required a comprehensive evaluation protocol as already described for the multicentre evaluation of BM/Hitachi 911 [2]. Seven European laboratories participated in the multicentre study in order to assess the analytical performance and practicability aspects of BM/Hitachi 917. Altogether, 43 different analytes covering 56 different methods—enzymes, substrates, electrolytes, specific proteins, drugs and urine applications—were tested in a core programme mainly following the ECCLS guidelines [3]. In addition, a specific satellite programme was carried out for specific tests with less extensive evaluation experiments in order to maintain an acceptable cost/benefit ratio. In total, more than 120 000 individual data were generated and statistically evaluated within a period of 7 months. Processing and analysis of the large data volumes were managed with the programme package CAEv (computer-aided evaluation). CAEv [4] allows the definition of protocols, the sample and test requests for on-line data capture, and statistical evaluation of results. Data were validated by the laboratories and sent via telecommunication to the central study administration.

### Description of the instrument

BM/Hitachi 917 is a selective access analyser with a capacity of at least 48 different tests onboard out of 86 stored applications including three ISE methods. The number of onboard tests can be increased by monoreagents which are distributed on any of the two reagent disks. The theoretical test throughput is 1200 tests per hour. Certain instrument conditions, e.g. predilution, high sample volume pipetting, mixing of short-long-term applications, STAT requests or additional wash steps for the pipettors or the cuvettes needed to eliminate reagent carry-over in certain cases lead to a reduction of the throughput. The pipetting cycle for photometric tests is 4.5 s and for the three ISE assays 18 s. The software has integrated an algorithm for throughput optimization. It recognizes pipetting conflicts—e.g. R2/R3 pipetting—and reschedules the steps so that the additional time needed for the conflict situation is a minimum. The bar-coded system reagents consisting of one–three vials per test are set in any free position of the reagent disk. For frequently requested tests, several bottles of one reagent can be loaded into the reagent disk. An automatic bottle changeover occurs after the first bottle is registered as empty. Application settings are loaded from application floppy disk or from application bar code sheet, both are delivered by Roche Diagnostics GmbH. Five applications are user definable. At present, over 200 applications are available.

Specimens are processed either from primary tubes (5–10 ml), secondary cups (2 ml) or microcups (0.5 ml) positioned on a sample disk with 110 positions. Primary tubes can be identified by four different types of bar codes with the possibility of mixing. A standardized RS232 interface allows a bidirectional communication to a host computer.

One hundred and sixty semi-disposable plastic cuvettes are arranged on a rotor positioned in a waterbath of 37 °C. The cuvettes pass through the beam of the photometer every 18 s; 12 fixed wavelengths between 340 and 800 nm in mono- or bichromatic mode can be selected.

Two pipettors transfer the reagent into a cuvette. The average reagent consumption is ~200 µl per determination. Most of the photometric STAT results are available 10–12 min after test request. Various measurement and calibration procedures can be applied. The main specifications of BM/Hitachi 917 are summarized in table 1.

### Materials and methods

### *Instruments and reagents*

The methods and instruments used in this study are listed in table 2. The same reagents were used in all evaluation centres for each method on BM/Hitachi 917. The reagents were available in special system packs designed for BM/Hitachi 917. For the comparison experiments the methods and reagent lots from the routine were used.

### *Calibration*

During the familiarization period, a fixed factor was determined for the enzyme assays in three independent calibration runs per day on three consecutive days. The same lot of the calibrator for automated systems (Roche Diagnostics GmbH) was used for this purpose. The fixed factor is the median from the median factor of the three calibration runs per day, provided that the coefficient of variation (CV ) calculated from the nine results is less than 3%.

The substrate, specific protein and drug assays were performed with the autocalibration which is triggered by an analyte-dependent calibration interval. For this reason, the respective calibrator material was placed in the cooled sample disk S2 of the instrument. The type of calibration and the autocalibration data for all analytes were predefined by Roche Diagnostics GmbH in the chemistry parameter settings, stored on the application floppy disk.

The immunoglobulins A,G,M, transferrin and C-reactive protein assays were calibrated according to CRM† standardization. The ISE methods were calibrated daily with the ISE standards and compensator. Detailed information about the calibrator materials employed is shown in table 9 .

### *Control materials*

Imprecision and quality control experiments were performed with lyophilized or liquid control sera from Roche Diagnostics GmbH and control urines from Roche Diagnostics GmbH and BioRad Laboratories; details are shown in table 9.

For accuracy testing standard reference materials, e.g. CRM‡ material for four IFCC enzyme methods and material from NIST§ for several substrate and electrolyte methods were used (table 9, details available on request).

A uniform procedure was applied to the treatment of lyophilized calibrator and control material in order to minimize matrix effects and stability problems. The materials were reconstituted within 30 min and then stored in the dark for a further 30 min before starting the calibration runs of the experiments.

### Evaluation protocol

The protocol for the analytical performance of the BM/Hitachi 917 analysis system comprised the testing of the quality characteristics within-run and between-day imprecision, analytical range limits, drift over 8 h, carry-over, interferences and accuracy based on recovery in control materials and method comparison. Forty-three different analytes covering 56 different methods—enzymes, substrates, electrolytes, specific proteins, drugs and urine applications—were tested (table 2).

The total versatility of the new analysis system was covered by a common core programme and by laboratory-specific satellite programmes. The core programme comprised 17 analytes from the classical field of clinical chemistry and was divided into two groups consisting of five laboratories each of which processed the same set of

† CRM 470 [5], BCR information, Community Bureau of Reference, Brussels.
‡ BCR information, Community Bureau of Reference, Brussels.
§ National Institute of Standards & Technology, Office of Standard Reference Materials, Gaithersburg, USA.

*Table 1. Instrument specifications.*

| | |
|---|---|
| (1) Type of instrument | Discrete selective multianalyser. |
| (2) Test channels | Forty-five, with ISE module 48, to be increased with monoreagents. |
| (3) Test procedures | Endpoint, endpoint with sample blank, kinetic with serum or substrate start with or without sample blank, fixed-time kinetic, combination of two endpoint tests, endpoint and kinetic tests, two kinetic tests performing two tests in one cuvette, two prozone check procedures, measurement with ISE, linear calibration and five non-linear modes of calibration, autocalibration, one- and two-point recalibration, isoenzyme calibration, serum indices indicating haemolytic, icteric and lipaemic specimens. |
| (4) Throughput | Maximum 800 photometric tests/h, with ISE-module 1200 tests/h. |
| (5) Sampling system | Sample disk 1 with 110 positions arranged in two concentric rings for routine and STAT samples in bar code identification mode. Up to 55 positions in the inner ring are user definable for STAT samples in non-bar code mode. Sample disk 2 with 57 cooled positions for calibrators and controls, and three fixed positions for wash solutions.<br>Primary tubes from 13 to 16 mm diameter and 75 to 100 mm length, secondary sample cups with 2.0 ml and microcups with 0.5 ml maximum volume. Bar code identification of primary tubes using codabar NW 7, code 39, two out of five interleaved and code 128. Different codes and tube sizes are allowed within one sample disk. |
| (6) Sample pipettor | Two–thirty-five microlitres (in steps of 0.1 µl), imprecision <1%; for ISE 15 µl for the three determinations of Na, K, Cl. |
| (7) Reagent cooling | Cold water circuit, refrigerator temperature 5–12 °C. |
| (8) Reagent bottles | Seven, 20 and 70 ml sizes, two reagent disks with 45 bottle positions. Disk 1 for reagent 1, disk 2 for reagent 1 (monoreagents), 2 and 3. |
| (9) Reagent dispenser | Two reagent pipettors for dispensing reagents 1–3, 20–270 µl steps in 1 µl steps. |
| (10) Mixing procedure | Two stirrers mix the reaction solution independently after addition of each reagent with additional mixing programmable. |
| (11) Reaction rotor | Turntable with 160 cuvettes; quarter rotation (37 cuvettes +4) in 4.5 s = one working cycle. |
| (12) Reaction cuvettes | Special plastic cuvettes, semidisposable. Volume required: minimum 180 µl, maximum 380 µl. Optical path length: 5 mm. |
| (13) Reaction cycle | Ten reaction times between 1 and 10 min corresponding to measuring points between 4 and 34. |
| (14) Temperature control | Waterbath, 37 ± 0.1 °C |
| (15) Photometer | Single-beam photometer with 12 available wavelengths: 340, 376, 415, 450, 480, 505, 546, 570, 600, 660, 700 and 800 nm; mono- or bichromatic measurement with free selectable wavelength combinations.<br>Light source: halogen lamp. Detector: silicone photodiode.<br>Wavelength adjustment: fixed, via a grating chromator, inaccuracy at 340 ± 2 nm and at 405–800 ± 55 nm.<br>Half band width: 4 nm in the UV range,<br>10 nm in the visible range.<br>Photometric range of linearity: $A = 0$–3.0 at 340 nm.<br>Photometric resolution: $A = 0.0002$.<br>Photometric inaccuracy: max. 1% at 2.0 abs. |
| (16) Ion-selective electrodes | Indirect potentiometry; flow-through electrode with liquid membrane.<br>Reference electrode, liquid membrane.<br>Dilution ratio: 1:31.<br>Incubation temperature: 37 ± 0.1 °C.<br>Calibration: two-point with compensation.<br>Measuring cycle: 18 s.<br>Measuring range — in serum — in urine<br>Na+ 80–180 — 10–250 mmol/l<br>K+ 1.5–10 — 1–100 mmol/l<br>Cl− 60–120 — 10–250 mmol/l<br>Consumption of ISE solution per sample:<br>Diluent: 450 µl<br>Internal standard: 1050 µl<br>KCl solution: 130 µl. |
| (17) Data processing | Data input: via touch screen or alpha numeric keyboard item select keys.<br>Data control: CRT 14 inch colour monitor.<br>Data output: matrix printer, 80 characters per line, 220 characters per second.<br>CPU with 16 MB RAM, 270 MB HD and 1 FD drive, 3.5 inches.<br>Interface: RS 232 C. Bidirectional link with a host computer. |
| (18) Water supply | Internal reservoir with an external supply.<br>Quality: <1 s.<br>Consumption during operation: 40 l/h |
| (19) Ambient temperature | 15–32 °C. |
| (20) Relative humidity | 45–85%. |
| (21) Physical dimensions | Analyser unit — Operation unit<br>Width 1.40 m — 0.65 m<br>Depth 0.77 m — 0.85 m<br>Height 1.17 m — adjustable. |
| (22) Weight | Approx. 400 kg. |

*Table 2. Analytes, methods and comparison instruments.*

| | *Methods on BM/Hitachi 917* | | *Comparison instruments and methods different from H917* | | | | | | | *Study units* |
|---|---|---|---|---|---|---|---|---|---|---|
| | | | *A* | *B* | *D* | *E* | *GB* | *I* | *NL* | |
| ALAT | Alanine aminotransferase | IFCC, with PYP | H717 opt. | H747 no PYP | H747 opt. | H747 | | | Chem I | U/l |
| ASAT | Aspartate aminotransferase | IFCC, with PYP | | H747 no PYP | H747 opt. | | H737 opt. | H747 | Chem I | U/l |
| ALP_I | Akaline phosphatase | IFCC | | | | H747 | | | | U/l |
| ALP_O | Alkaline phosphatase | DGKCh | | H747 | | | | | | U/l |
| AMYLP | Pancreatic $\alpha$-amylase | EPS | | H747 CNP malt. | H747 | | H717 | | Chem I | U/l |
| AMYLT | Total $\alpha$-amylase | EPS | | | | | | H747 | | U/l |
| CK | Creatine kinase | NAC activ. | | H747 | H747 | | C. FARA Sigma | H747 | ELAN | U/l |
| CK-MB | Creatine kinase, MB-isoform | Immuninhibition | | | | | C. FARA Sigma | | | U/l |
| GGT_I | $\gamma$-Glutamyl-transferase | IFCC | | | | H747 | | | | U/l |
| GGT_S | $\gamma$-Glutamyl-transferase | Szasz | H717 | H747 | H747 | | | | Chem I | U/l |
| LDH_O | Lactate dehydrogenase | DGKCh | H717 | | H747 | | | | Chem I | U/l |
| LDH_S | Lactate dehydrogenase | SFBC | | H747 | | H717 | | | | U/l |
| LIP | Lipase | Turbidimetric | H717 | | | | | | | U/l |
| CHOL | Cholesterol | CHOD-PAP | | H747 | H747 | | H717 | H747 Biotr. Abel Kend. | Chem I | mmol/l |
| CREAJ | Creatinine | Jaffé | H717 | H747 | | H747 | | | Chem I | µmol/l |
| CREAP | Creatinine | PAP | | | H747 | | | | | µmol/l |
| GLU_HK | Glucose | HK | | H747 | H747 | | C. FARA | H747 | Dimens. | mmol/l |
| TP | Total protein | Biuret | | H747 | H747 | | H737 | H747 | Chem I | g/l |
| UA | Uric acid | PAP | H717 | H747 | H747 | H747 | | | Chem I | µmol/l |
| UREA | Urea | UV | H717 | H747 | H747 | H747 | | | Chem I | mmol/l |
| CA | Calcium | OCPC | | H747 | H747 | | H717 | H747 | Chem I | mmol/l |
| FE | Iron | Ferrozine | H717 | H747 | H747 | H747 | | | EPOS | µmol/l |
| LACT | Lactate | UV | | H911 PAP/Biom. | | | | | | mmol/l |
| NA_I | Sodium | ISE | H717 | H747 | H747 | H747 | H737 | H747 | Chem I | mmol/l |
| K_I | Potassium | ISE | H717 | H747 | H747 | H747 | H737 | H747 | Chem I | mmol/l |
| CL_I | Chloride | ISE | H717 | H747 | H747 | H747 | H737 | H747 | Chem I | mmol/l |
| APOA1 | Apolipoprotein A1 | TIA, IFCC | | | | H717 | | BNA | Array | g/l |
| APOB | Apolipoprotein B | TIA, UFCC | | | | H717 | | BNA | Array | g/l |
| ASLO | Antistreptolysin O | LPIA | BN-II | | | | | | | IU/ml |
| CRP | C-reactive protein | TIA | H717 | H911 | BNA BNA | H717 | FLX Abbott | BNA | Dimens. | mg/l |
| DDIM | D-Dimer | LPIA | Nycocard | | | | | Sclavo | | mg/l |
| FERRI | Ferritin | LPIA | C. Core | | ES 700 | | | | ES 600 | µg/l |
| HBA1C | Haemoglobin A1c | TIA | HPLC Shimatsu | H911 | | | | | HPLC Biorad | % |
| IGA | Immunoglobulin A | TIA | | BNA | | | | | Array | g/l |
| IGG | Immunoglobulin G | TIA | | BNA | | | | | Array | g/l |
| IGM | Immunoglobulin M | TIA | | BNA | | | | | Array | g/l |
| RF | Rheumatoid factor | LPIA | BNA | BNA | | H717 | | | | IU/ml |
| TRANS | Transferrin | TIA | H717 | H911 | BNA | H717 | | | Array | g/l |
| DIG | Digoxin | LPIA | H717 | | | | | STRATUS/ ELAN | | µg/l |
| DIGIT | Digitoxin | CEDIAR assay | H717 | | | | | | | µg/l |
| PHEBA | Phenobarbital | CEDIAR assay | | | | | | ELAN SYVA | | mg/l |
| THEO | Theophylline | CEDIAR assay | | | | | | ELAN SYVA | | mg/l |
| T4 | Thyroxine | CEDIAR assay | | | | | ACS CIBA CORN. | | | nmol/l |
| A1MG | $\alpha$-1 Microglobulin | TIA | | | — | | | | | mg/l |
| MAU | Albumin in urine | TIA | | RIA | — | | | | Array | mg/l |
| CREAJU | Creatinine (in urine) | Jaffé | | H911 | | | C. FARA | | EPOS | mmol/l |
| CREAPU | Creatinine (in urine) | PAP | NOVA | | H747 | | | | | mmol/l |
| GLUHKU | Glucose (in urine) | HK | | H911 | H747 | | | | EPOS | mmol/l |
| UA_U | Uric acid (in urine) | PAP | | H911 | H747 | | | | EPOS | µmol/l |
| UREA_U | Urea (in urine) | UV | NOVA | H911 | H747 | | H737 | | EPOS | mmol/l |
| CA_U | Calcium (in urine) | OCPC | | H911 | H747 | | H717 | | EPOS | mmol/l |
| MG_U | Magnesium (in urine) | Xylidyl blue | | | H747 | | H717 | | AAS | mmol/l |
| PHOS_U | Phosphate (in urine) | Molybdate, UV | | H911 | H747 | | H717 | | EPOS | mmol/l |
| NA_IU | Sodium (in urine) | ISE | NOVA | H911 | H747 | | Flame | | Dimens. | mmol/l |
| K_IU | Potassium (in urine) | ISE | NOVA | H911 | H747 | | Flame | | Dimens. | mmol/l |
| CL_IU | Chloride (in urine) | ISE | NOVA | H911 | H747 | | Flame | | Dimens. | mmol/l |

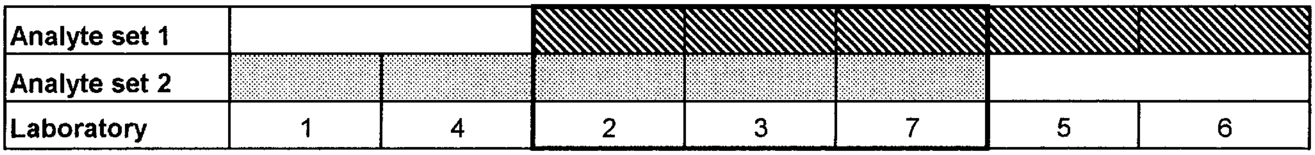

*Figure 1. Contribution of analytes of the core programme over the evaluation sites.*

analytes. Three laboratories took all analytes of the core programme (figure 1). The protocol was designed in that way where each analyte was processed in an odd number of laboratories so that the median of the statistics from the individual laboratories is related to the outcome of a single experiment. The ISE analytes were performed by all evaluation sites. The various analytes were split between the seven evaluation sites for the studies of linearity, drift and sample-related carry-over. Similarly, the testing for endogenous interferences was shared between the laboratories. Only the core programme covered the total evaluation phase with familiarization, initial trial and main trial. The initial trial consisted of a between-day imprecision experiment over 11 days. The evaluation protocol of the main trial is shown in table 3.

The satellite programme contained analytes from various laboratory segments, e.g. specific proteins, drugs and urinalysis. This programme was integrated into the main trial and included the experiments within-run and between-day imprecision, method comparison and in most cases analytical range limits and interference.

During a start-up meeting of the multicentre evaluation, all evaluators agreed upon the protocol and the quality specifications proposed by Boehringer Mannheim.

### Software upgrade evaluation

The total evaluation of the analytical performance was carried out with software version V1. In addition to this evaluation a software functionality testing of the new version V2 was performed. This new version includes improvements of certain screen designs and new functions, e.g. enhanced data management capabilities, bar code sheets for convenient transfer of applications, calibrator and control material information, reagent exchange during operation, a new quality control package, usage of monoreagents either on reagent disk one or two in order to increase the number of tests on board, and a context sensitive help system.

The evaluation protocol comprised a familiarization phase with the new software, a within-run imprecision experiment with two control sera and a human specimen pool to provide the information that the new software version shows a comparable imprecision. Routine simulation experiments related to reproducibility and download experiments to test comparability and functionality testing of the bar code sheets for applications, calibrators and control materials should prove reliability and correct system functionality.

As for the other experiments of the study, the definition and performance of the routine simulation was carried out with the software package CAEv [10]. Reproducibility was tested in an experiment based on the within-run imprecision concept which consisted of two parts, a 'reference' part being performed as a usual imprecision run with two control materials and at least two human specimen pools in 15 repetitions, followed by the random part with variable numbers of requests (1–23) per sample and variable test pattern per sample type according to the routine situation of the laboratory [1]. In a second simulation imprecision experiment, provocation steps to the analytical system were integrated, e.g. sample short, STAT sampling, reagent interrupt, reagent bar code error, sample bar code error, additional test selection.

In two routine download experiments, ~100 samples from the daily routine runs were transferred to the BM Hitachi instrument. The sample sequence and the results were downloaded to the CAEv database, and a corresponding request for BM/Hitachi was generated [1].

### *Assessment of reliability and practicability*

For the assessment of reliability, a logbook was kept throughout the total evaluation period (7 months, multicentre evaluation and software V2 testing). Any breakdown, defect, malfunction or unexpected incident of the analysis system was recorded.

Practicability was assessed with the aid of a questionnaire [11] comprising ~200 questions or attributes which covered all important aspects of an analysis system in the clinical laboratory. The attributes were summarized into 14 groups, as shown in figure 7. They were related to the installation of the analyser, organization of work, quality assurance and miscellaneous characteristics.

The assessment was based on a scale from 1 to 10 for the instrument under evaluation as well as for the present laboratory situation. A score of 1 meant unimportant, useless or poor, and a score of 10, absolutely necessary or excellent. The meaning of score 5 was acceptable or comparable with the present laboratory situation. Additionally, a weight factor was assigned to each of the attributes. The factor ranged from 0 to 3 with the following meanings: 0, the attribute was not used during this assessment; 1, the attribute was unimportant for the laboratory; 2, the attribute was of general importance for the assessment; and 3, the attribute is very important for the evaluation site.

*Table 3. Evaluation protocol.*

*Imprecision*

Within-run
On 3 days, each day one run with 21 aliquotes.

- Two control materials (serum, plasma, urine) with different concentrations of the analyte.
- One human specimen pool at the decision level.

Between-day

- Two control materials with different concentrations of the analyte and one human pool (deep frozen and thawed) at the decision level over 11 days and subsequent 10 days in the main trial combining the two parts into one experiment. Precision is derived from the second of triplicate measurements.

Drift

- Two control sera and the calibrator are determined every 30 min during 8 h.
- At zero hour the base value is determined as the median of triplicate measurements.
- The percentage recovery from the base value is taken as the measure for drift effects.

Linearity

Protocol is based on Ref. [3].
Mixing of a high level with a low level specimen leads to:

- a dilution series of 11 concentration steps with nine dilution steps plus two basic concentrations;
- triplicate measurements of samples from the 11 concentration steps and calculation of the median for each step;
- calculation of the regression line (P/B-regression [7] using values of five concentrations, the range of which is assumed to be linear;
- calculation of the target values for all concentration steps from the regression line.

Carry-over

Sample-related
Model of Broughton [8]

- Measurements of five aliquots of a high-concentration sample $(h_1 \ldots h_5)$.
- Followed by measurements of five aliquots of a low-concentration sample $(l_1 \ldots l_5)$.

The experiments are repeated 10 times.
If a carry-over effects exists, the $l_1$ is the most influenced, $l_5$ the least influenced aliquot.
The sample-related carry-over—median $(l_1–l_5)$—is compared with the imprecision of the low-concentration sample.

Reagent-dependent

Assay A influences assay B.

- Carry-over caused by the cuvettes.
  Test A is pipetted into 21 cuvettes and the analyser is stopped. Assay B is performed in 42 cuvettes; the first 21 determinations may be influenced by assay A, the last 21 determinations are uninfluenced. The difference of the medians of both series is the carry-over.
- Carry-over caused by reagents probes and stirrers.
  Assay B is carried out 21 times. In a second step, test A and B are requested 21 times. The carry-over is the difference between the medians of both series.
  The carry-over effects are compared with the imprecision and the diagnostic relevance of assay B.

Interference

Protocol of Glick [9].
A serum with concentrations at the relevant decision level is spiked with the interfering substance, and a dilution series of 10 dilution steps is prepared with the same baselinee serum. The different analytes are measured in triplicate. The concentration of the interfering substance is related to the serum index of the instrument. The percentage recovery of the baseline value from the corresponding analyte is calculated for each dilution step.
The serum indices characterize the specimens according to haemolytic, icteric and lipaemic interference. The index for bilirubin and haemoglobin corresponds approximately to the concentration of these interferents, and the lipaemic index is related to the turbidity at 660/700 nm expressed at absorbance × 10.000.

Accuracy

Calibration.

- The calibrators of BM/Hitachi 917 and of the comparison instrument are both run on each instrument.

Quality control in two control materials.

- Assigned values for several substrate and electrolyte methods are related to reference methods.
- Median, calculated from the second of triplicate measurements on 21 days.

Interlaboratory survey.

- One control material with concentrations not known to the evaluators; assigned values for several substrate methods are related to reference methods.
- Median, calculated from the second of triplicate measurements over 10 days.

Standard reference material.

- For certain enzymes, substrate and electrolyte methods analysed on 1 day in triplicate measurements.

Method comparison in fresh human specimens.

- Five–15 specimens pr day depending on analytes for 10 days on BM/Hitachi 917 and on the comparison instruments. The total number of specimens cover the entire analytical range.
- Comparison of the methods by calculation of the Passing/Bablok regression line [7].

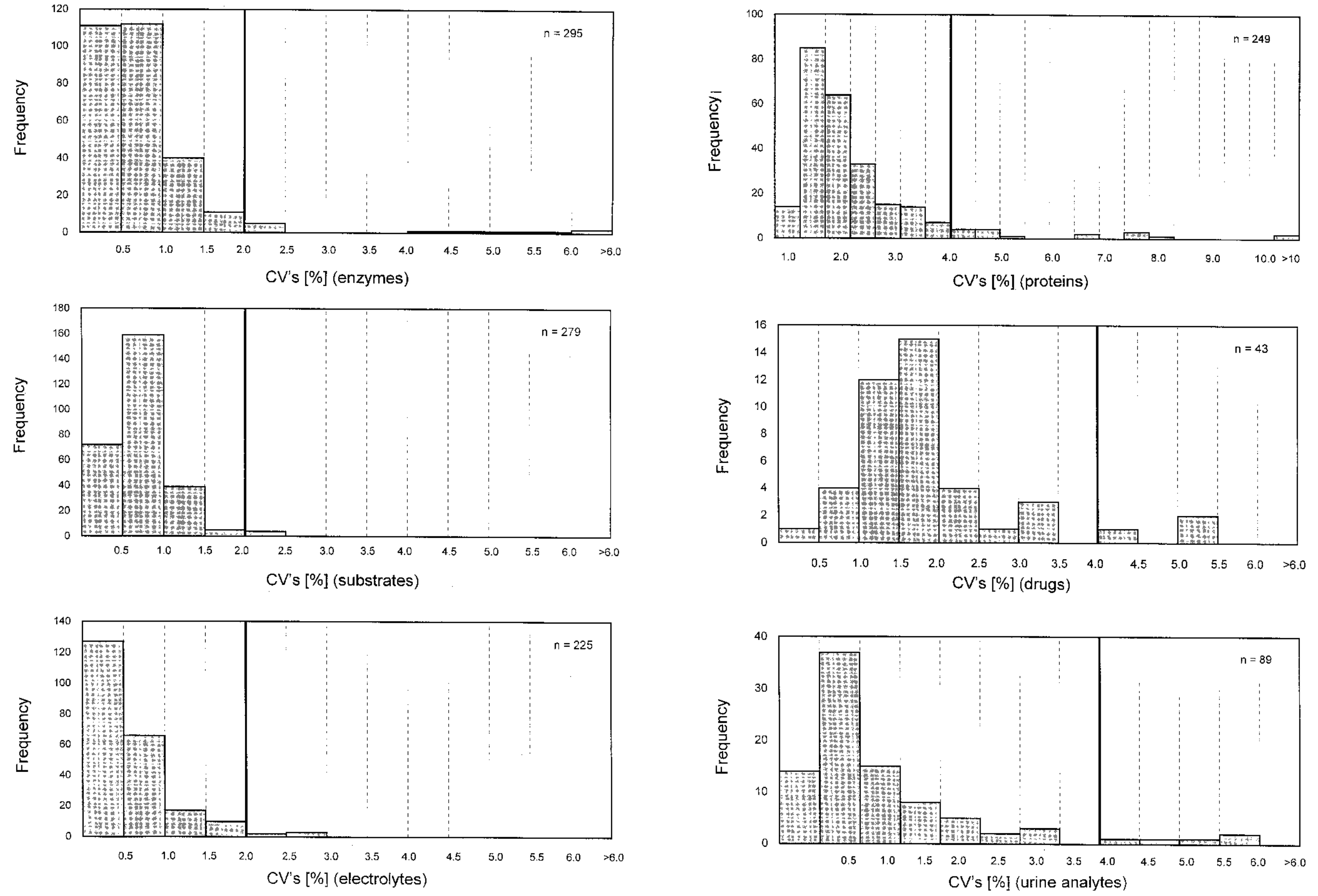

*Figure 2. Within-run imprecision, frequency distribution of all CVs.*

### Quality specifications

The agreed acceptance criteria for imprecision are set up with a view to fulfilling requirements of the daily laboratory routine and statistical error propagation [12]; they are listed in table 4. Additionally, imprecision is judged on criteria based on within-subject biological variation according to Fraser *et al.* [13, 14]

The quality specifications for the within-run CV of the enzyme and substrate methods are derived from error propagation as shown in Ref. [12]. Due to the daily variation of the analysis system, one should expect a higher CV compared to the within-run CV. The ISE methods in general show a better reproducibility than the photometric assays. Drug and specific protein assays have very often low analytical sensitivity and many of them are calibrated by a non-linear mode; therefore, a CV twofold higher than that of the classical photometric determinations is reasonable. Because urine applications are performed in several cases with a sample predilution and are calibrated with the serum application volume ratio, which is not adequate for urine concentrations, an elevated CV can be expected.

*Table 4. Acceptance limits for imprecision.*

| *Analyte group* | *Within-run CV(%)* | *Between-day CV(%)* |
|---|---|---|
| Enzymes | 2 | 3 |
| Substrates | 2 | 3 |
| ISE methods | 1 | 2 |
| Specific proteins | 4 | 6 |
| Drugs | 4 | 6 |
| Urine methods | 4 | 6 |

The measuring range of a method should cover the greatest part of the physiological and pathophysiological range so that rerun analyses rarely will be necessary. In the upper range, a method is defined to be linear if the differences between the measured values and the target values from the dilution series are below 5%. In the lower range, the absolute differences are judged with respect to the diagnostic relevance. Methods with multipoint calibration are regarded as linear if a change in the target concentration leads to a corresponding change in the measured concentration [6].

Drift effects are not accepted if a systematic deviation from the initial value exceeds 3%.

Carry-over effects are assessed on the basis of the observed change in recovery of an analyte. Instead of adapting an individual deviation for each analyte, the within-run imprecision system performance is used which

*Table 5. Within-run imprecision in a normal human serum, plasma or urine pool* ($n = 21$).

| *Analyte* | *Unit* | *Concentration* | *CV (%)* |
|---|---|---|---|
| Alanine aminotransferase | U/l | 49.4 | 0.5 |
| Aspartate aminotransferase | U/l | 44.9 | 1.0 |
| Alkaline phosphatase | U/l | 102 | 0.5 |
| $\alpha$-Amylase total | U/l | 168 | 1.0 |
| $\alpha$-Amylase pancreatic | U/l | 79.2 | 1.1 |
| Creatine kinase | U/l | 163 | 0.6 |
| Creatine kinase MB | U/l | 14.4 | 5.8 |
| $\gamma$-Glutamyltransferase | U/l | 42.2 | 1.2 |
| Lactate dehydrogenase | U/l | 446 | 0.3 |
| Cholesterol | mmol/l | 5.3 | 1.0 |
| Creatinine | µmol/l | 115 | 0.9 |
| Glucose | mmol/l | 6.32 | 1.0 |
| Total protein | g/l | 68.8 | 1.0 |
| Uric acid | µmol/l | 300 | 0.6 |
| Urea | mmol/l | 8.65 | 0.7 |
| Calcium | mmol/l | 2.33 | 0.6 |
| Iron | µmol/l | 13.7 | 2.0 |
| Lactate | mmol/l | 2.19 | 0.4 |
| Sodium | mmol/l | 143 | 0.4 |
| Potassium | mmol/l | 4.63 | 0.3 |
| Chloride | mmol/l | 106 | 0.9 |
| Apolipoprotein A-1 | g/l | 1.29 | 1.4 |
| Apolipoprotein B | g/l | 1.05 | 0.5 |
| Antistreptolysin O | IU/ml | 85 | 1.8 |
| D-dimer | mg/l | 1.5 | 1.5 |
| C-reactive protein | mg/l | 7.6 | 3.0 |
| Ferritin | µg/l | 77.6 | 1.7 |
| Haemoglobin $A_{1c}$ | % | 5.94 | 2.8 |
| Immunoglobulin A | g/l | 2.8 | 1.0 |
| Immunoglobulin G | g/l | 11.27 | 1.4 |
| Immunoglobulin M | g/l | 1.19 | 0.8 |
| Rheumatoid factor | IU/ml | 22.9 | 3.5 |
| Thyroxine | nmol/l | 104 | 2.2 |
| Transferrin | g/l | 3.2 | 0.7 |
| Digoxin | µg/l | 0.74 | 4.5 |
| Digitoxin | µg/l | 17.9 | 3.1 |
| Phenobarbital | mg/l | 24.6 | 0.8 |
| Theophylline | mg/l | 7.4 | 1.7 |
| Albumin in urine | mg/l | 16.3 | 3.0 |
| $\alpha$-1-Microalbumin | µg/l | 7.2 | 2.2 |
| Creatinine in urine | mmol/l | 13 | 0.6 |
| Glucose in urine | mmol/l | 17.2 | 0.8 |
| Uric acid in urine | µmol/l | 2.5 | 1.4 |
| Urea in urine | mmol/l | 283 | 0.9 |
| Magnesium in urine | mmol/l | 3.7 | 1.4 |
| Sodium in urine | mmol/l | 43 | 0.5 |
| Potassium in urine | mmol/l | 68 | 0.3 |

means that a change of less than twice the standard deviation is accepted.

According to Glick *et al.* [9], a method is resistant to interference if the deviation between the baseline value and the measured value is less than 10%.

Accuracy is assessed in recovery experiments performed with certified reference materials, with control materials of which the assigned values are related to reference methods, and in method comparison experiments in which the comparison method is a reference method. A relative accuracy is obtained by the usual method comparison experiments related to the routine methods of the laboratory.

Results of the recovery experiments are defined as being acceptable if their deviations from the target values do not exceed more than ± 5% for enzymes and substrates, and ±3% for the electrolyte methods calcium, chloride, potassium and sodium.

For the method comparisons, the acceptable range is defined for the slope and intercept of the regression equation. The slope should not deviate more than ±5% from the identity line and the intercept should

*Table 6. Linearity.*

| *Analyte/Method* | *Unit* | *Lab* | *Concentration tested* | *Linearity found* |
|---|---|---|---|---|
| Alanine aminotransferase | U/l | 3 | 270 | 270 |
| | | BM* | 440 | 310 |
| Aspartate aminotransferase | U/l | 3 | 340 | 340 |
| | | BM* | 550 | 550 |
| Alkaline phosphatase/IFCC | U/l | 4 | 770 | 770 |
| | | BM* | 2500 | 2500 |
| $\alpha$-Amylase total | U/l | 6 | 1500 | 1500 |
| | | BM* | 3200 | 3200 |
| $\alpha$-Amylase pancreatic | U/l | 7 | 2100 | 2100 |
| Creatine kinase | U/l | 2 | 1750 | 1750 |
| Creatine kinase MB | U/l | 5 | 230 | 230 |
| $\gamma$-Glutamyltransferase/IFCC | U/l | 4 | 900 | 900 |
| | | BM* | 1600 | 1600 |
| $\gamma$-Glutamyltransferase/Szasz | U/l | 7 | 1200 | 1200 |
| | | BM* | 1800 | 1800 |
| Lactate dehydrogenase/opt. | U/l | 3 | 1060 | 1000 |
| | | BM* | 1600 | 1300 |
| Lactate dehydrogenase/SFBC | U/l | 4 | 600 | 600 |
| | | BM* | 1900 | 1900 |
| Cholesterol | mmol/l | BM* | 22.9 | 22.9 |
| Creatinine | µmol/l | BM* | 2100 | 2000 |
| Glucose | mmol/l | 6 | 53 | 53 |
| Total protein | g/l | 5 | 115 | 115 |
| | | BM* | 190 | 190 |
| Uric acid | µmol/l | 1 | 1550 | 1550 |
| | | BM* | 1800 | 1800 |
| Urea | mmol/l | 1 | 44 | 44 |
| | | BM* | 70 | 70 |
| Calcium | mmol/l | 6 | 6.3 | 5.9 |
| Iron | µmol/l | BM* | 314 | 314 |
| Lactate | mmol/l | 2 | 16 | 15 |
| Sodium | mmol/l | 5 | 150 | 150 |
| | | BM* | 300 | 300 |
| Potassium | mmol/l | 5 | 6.3 | 6.3 |
| | | BM* | 15 | 15 |
| Chloride | mmol/l | 5 | 112 | 112 |
| | | BM* | 270 | 270 |
| Antistreptolysin O | IU/ml | 1 | 580 | 560 |
| C-reactive protein | mg/l | 4 | 180 | 180 |
| Ferritin | µg/l | 3 | 400 | 400 |
| Immunoglobulin A | g/l | 2 | 8.6 | 8.6 |
| Immunoglobulin G | g/l | 2 | 40 | 40 |
| | | BM* | 56 | 50 |
| Immunoglobulin M | g/l | 2 | 7.5 | 7.5 |
| | | BM* | 10 | 10 |
| Thyroxine | nmol/l | 5 | 248 | 248 |
| Transferrin | g/l | 3 | 3.2 | 3.2 |
| Digoxin | µg/l | 6 | 7.5 | 7.5 |
| Digitoxin | µg/l | 1 | 44 | 44 |
| Albumin in urine | mg/l | 3 | 370 | 370 |
| $\alpha$-1-Microalbumin | µg/l | 3 | 94 | 94 |
| Calcium in urine | mmol/l | 7 | 12 | 12 |
| Creatinine in urine | mmol/l | 7 | 32 | 32 |
| Uric acid in urine | µmol/l | 7 | 17 | 17 |
| Urea in urine | mmol/l | 7 | 950 | 950 |
| Sodium in urine | mmol/l | 7 | 220 | 220 |
| Potassium in urine | mmol/l | 7 | 93 | 93 |
| Phosphorus in urine | mmol/l | 7 | 100 | 100 |

* Evaluation data from Boehringer Mannheim.

be less than ±5% of the diagnostically important decision level. Due to the lower analytical sensitivity and the non-linear calibration mode of many of the homogeneous immunoassays, the acceptance limits for the method comparisons must be set higher. A deviation of ±10% for the slope is tolerated by the evaluators. Likewise, a deviation of ±10% from the decision or the detection limit is acceptable.

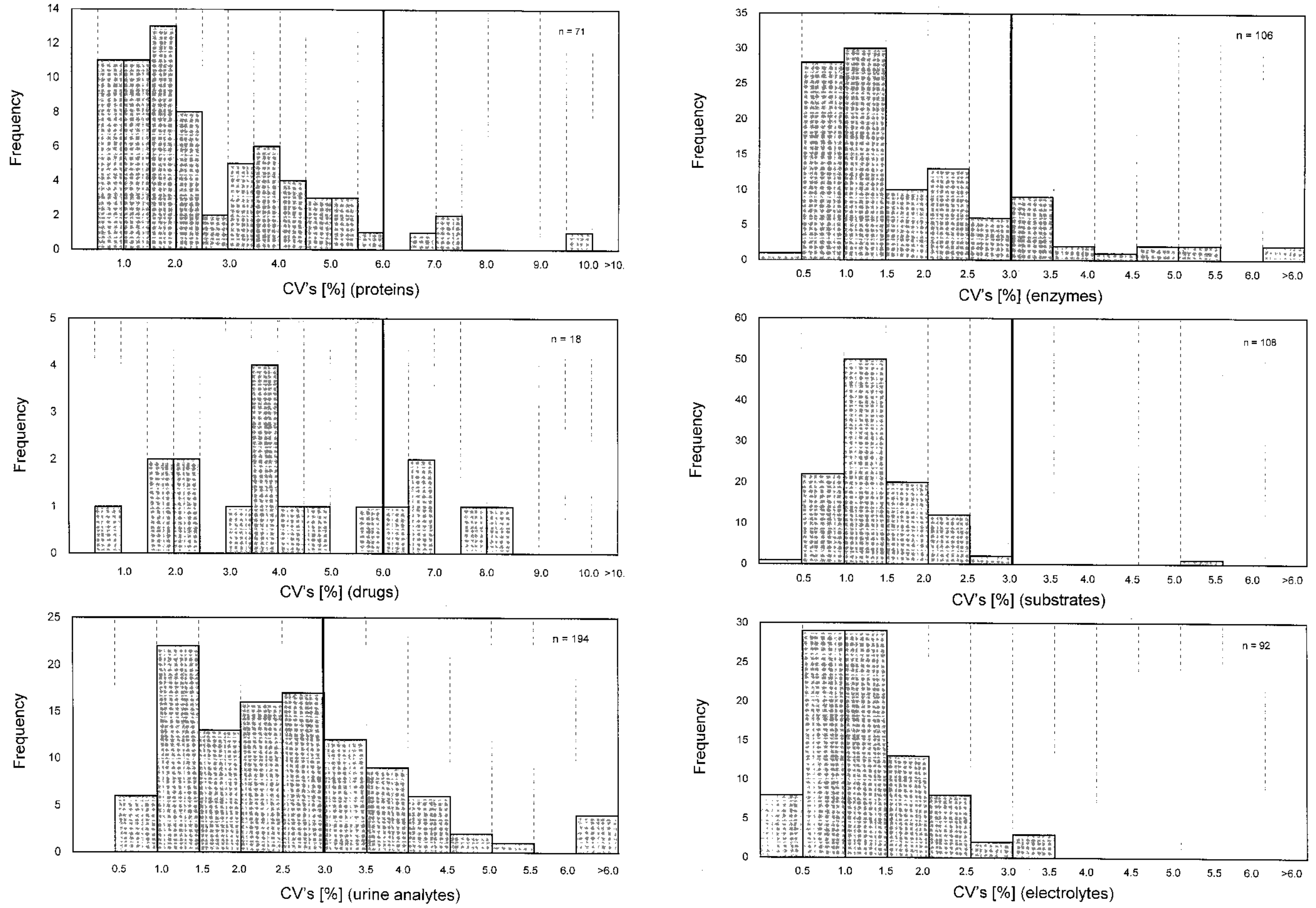

*Figure 3. Between-day imprecision, frequency distribution of all CVs.*

*Table 7. Sample related carry-over.*

| *Analyte* | *Unit* | *Lab* | *Median high conc.* | *Median low conc.* | *Ratio* $h:l$ | *Median* $I_1 - I_5$ | *SD* $I_3, I_4, I_5$ |
|---|---|---|---|---|---|---|---|
| Amylase total | U/l | 6 | 10263 | 96 | 107 | 0 | 1.1 |
| Creatine Kinase | U/l | 7 | 3606 | 55 | 65.6 | 1 | 0.6 |
| Ferritin | µg/l | 1 | 7300 | 17.8 | 410 | −0.3 | 0.5 |
| Ferritin | µg/l | 7 | >900 | 80 | >11 | 0.7 | 1.3 |
| Albumin U/S | mg/l | 3 | 40 000 | 27.2 | 1470 | 5.3 | 0.22 |
| Creatinine U/S | mmol/l | 3 | 15.2 | 0.12 | 127 | 0 | 0.003 |
| Creatinine U/S | mmol/l | 2 | 25.1 | 0.08 | 331 | 0 | 0.001 |
| Creatinine U/S | mmol/l | 5 | 20.1 | 0.1 | 201 | 0 | 0.003 |
| Creatinine U/S | mmol/l | 7 | 22.5 | 0.11 | 201 | 0 | 0.001 |
| Potassium U/S | mmol/l | 3 | 150 | 3.89 | 38 | 0.05 | 0.012 |
| Potassium U/S | mmol/l | 3 | 150 | 4.02 | 37 | 0.04 | 0.009 |
| Potassium U/S | mmol/l | 5 | 124 | 4.14 | 30 | 0.03 | 0.027 |

U = urine; S = serum.

Assessment of the ISE methods in serum or plasma cannot be achieved according to the above-mentioned criteria. Due to the narrow physiological range, especially of sodium and chloride, a relatively large confidence interval for the regression line is obtained. Therefore, method comparisons are judged by the concentration range in which the difference between the methods is less than 3%.

Sodium: 120–170 mmol/l.
Potassium: 2–10 mmol/l.
Chloride: 80–130 mmol/l.

### Results

### *Imprecision*

Acceptance criteria were based on statistical error propagation [12] (see table 4). Within-run distribution of all CVs measured for all analytes are shown in figure 2, additionally the median CVs for within-run imprecision in a human serum and urine pool are presented in table 5. The medians of all analytes met the acceptance criteria, except for creatine kinase MB isoform (CV of 5.8% in the human serum pool), lipase (CV of 5.2% in the human

*Table 8. Endogeneous interferences.*

| *Analyte* | *Haemolysis* | *Icterus* | *Lipaemia* |
|---|---|---|---|
| Alanine aminotransferase | ↑ | | |
| Aspartate aminotransferase | ↑↑↑* | ↑ | ↑ |
| α-Amylase total | ↑ | | |
| α_Amylase pancreatic | | | ↑ |
| Creatine kinase | ↑↑ | ↑ | |
| Creatine kinase MB | | ↑↑ | |
| γ-Glutamyltransferase | ↑↑ | ↑ | |
| Lipase | ↑↑ | | |
| Lactate dehydrogenase | ↑↑↑* | | |
| Cholesterol | | ↑ | |
| Creatinine PAP | | ↑↑↑ | |
| Total protein | ↑ | ↑ | |
| Uric acid | | ↑↑ | |
| Calcium | | ↑ | |
| Iron | ↑↑* | | |
| Potassium | ↑↑* | | |
| D-dimer | ↑↑ | | |
| C-reactive protein | | ↑ | |

* Present in erthyrocytes.

serum pool), digoxin (4.5% in the human serum pool) and albumin in urine (4.3%). In individual control sera results exceeding the acceptance limits were obtained for ALAT (2.1%), creatinine (2.4%), chloride (2.8%) and glycosylated haemoglobin (4.3%).

The distribution of all CVs measured in all sera for between-day imprecision are presented in figure 3. The medians met the defined quality specifications for the majority of analytes. The acceptance limits for median CV were exceeded for creatine kinase (3.3% in the human serum pool), creatine kinase MB isoform (9.4% in the human serum pool), lipase (4.5% in control serum 2 and 4.8% in the human serum pool), D-dimer (6.6% in control serum 1), glycosylated haemoglobin (6.9% in control serum 2), rheumatoid factors (9.8% in control serum 2), digoxin (7.8% in the human serum pool), digitoxin (6.6% in control serum 1 and 8.4% in the human serum pool), theophylline (6.7% in the human serum pool) and albumin in urine (up to 23% below 10 mg/l). In individual control sera, results exceeding the quality specifications could be seen for alkaline phosphatase (3.6%) and chloride (up to 3.2%).

Additionally, the data related to within-day imprecision are judged by the maximum allowable imprecision based on within-subject biological variation according to Fraser *et al.* [13, 14]. Median CVs for between-day imprecision were within these criteria for all analytes except for sodium (1.0%), chloride (1.6%), digoxin (8.4%) and phenobarbital (3.9%).

*Analytical range limit*

In table 6, the results of the assessment of linearity are presented. A wide linearity range was obtained for all clinical chemistry analytes, covering the greatest part of the clinical relevant range. All results were within the specifications of the manufacturer.

*Drift*

Drift effects were not accepted if a systematic deviation from the initial value exceeded 3%. No drift effects were observed over an 8 h period in any of the methods tested.

*Carry-over*

In table 7, the results of the sample carry-over testing are presented. If the carry-over is less than twice the standard deviation of the analyte tested, the results are judged as being acceptable. As can be seen from table 7, all results are acceptable except albumin in urine. The carry-over of 5.3 mg/l is not only exceeding twice the standard deviation (0.44 mg/l), but also the criteria of the manufacturer (1 mg/l). Considering the reagent-dependent carry-over, there was a significant probe carry-over in two out of seven laboratories (ALAT/LDH) and a cuvette carry-over (TG/LIP) in one out of seven laboratories. This could be explained by sub-optimal wash procedures in the analysers concerned.

*Interferences*

According to Glick *et al.* [9], a method is resistant to interferences if the deviation between the baseline value and the measured value is below 10%. The methods not fulfilling these criteria are presented in table 8.

*Accuracy*

The results of the recovery experiments in the certified reference materials are presented in figure 4. As can be seen, the results for enzymes (95.3–103.9%) are all within the 95–105% range. Considering the substrates and electrolytes there was a good performance except for creatinine, urea and chloride. Creatinine showed a recovery up to 120% in SRM 909 a-1 and a recovery of only 93% in SRM 909 a-2. The recovery for urea is only slightly above the tolerance limits (105.8%) and therefore not very relevant. The recovery for chloride is significantly different between the two laboratories concerned. This is possibly caused by a lot-to-lot variability.

The results for the recovery in the interlaboratory survey are presented in figure 5. The analytes outside the recovery limits for the reference materials also show results exceeding the limits in the interlaboratory survey. Additionally, results for alanine aminotranferase, aspartate aminotranferase, pancreatic amylase, cholesterol and glucose were not within the quality specifications for all laboratories.

*Method comparison*

In total, 160 method comparison studies were performed, using fresh human sera or urines; representative regression equations of each group of analytes are shown in figure 6. Additional regression data are available on request.

For cholesterol and creatinine, the results are compared to both routine and reference methods. As can be seen from figure 6, cholesterol meets the acceptance criteria if

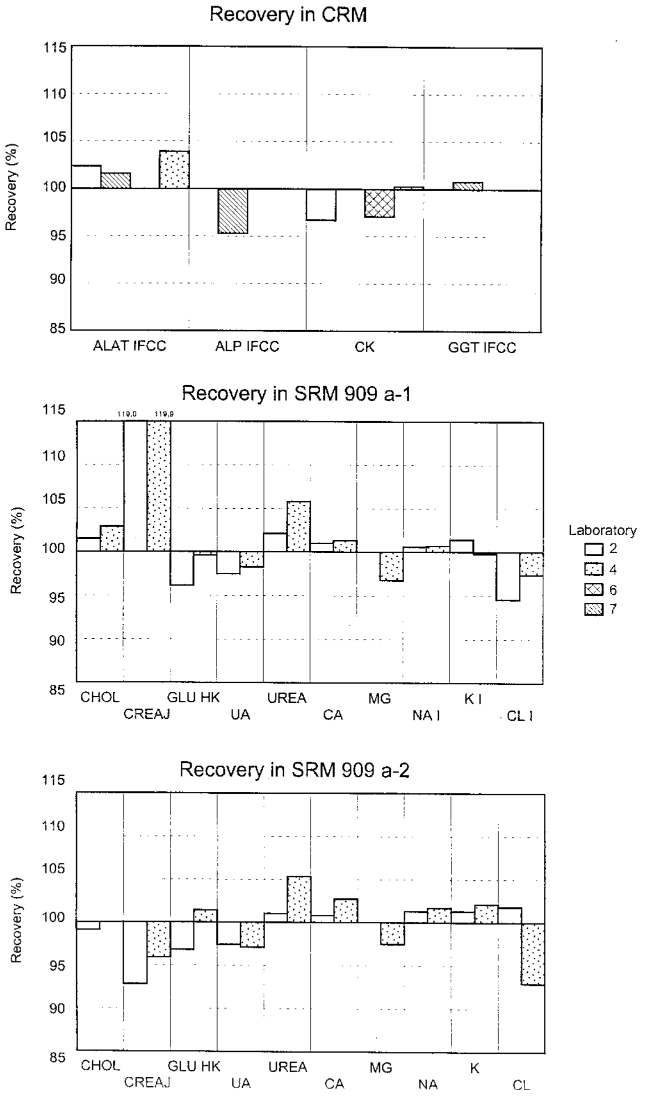

*Figure 4. Accuracy CRM/NIST.*

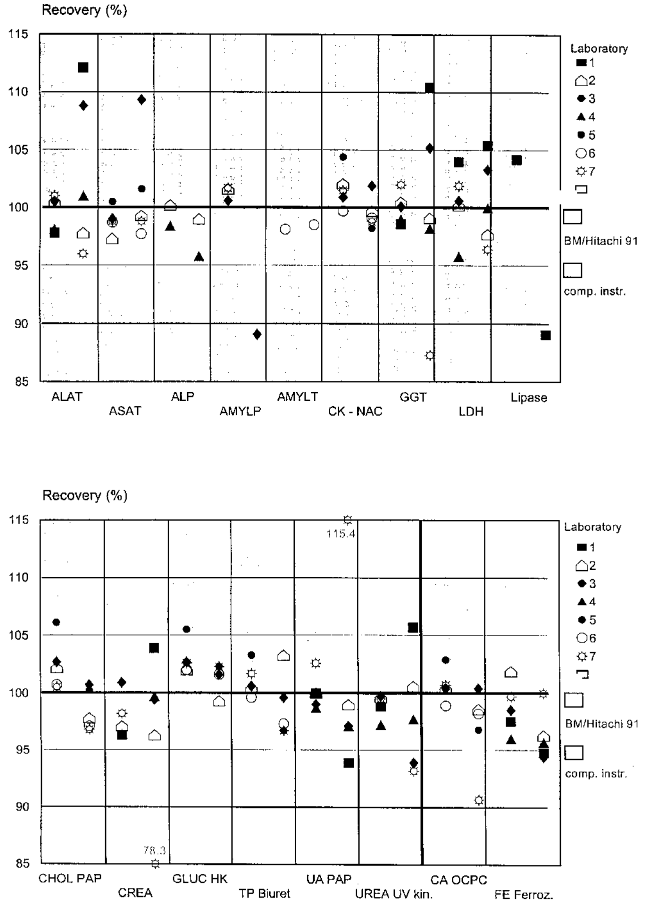

*Figure 5. Interlaboratory survey.*

compared to the Abell Kendall reference method, but creatinine on the BM/Hitachi 917 is inaccurate if compared to the HPLC reference method. For all other analytes, regression data do show an acceptable regression equation with the exception of ASAT, ALAT, sodium, chloride and glycosylated haemoglobin in some laboratories.

### *Reliability*

Reliability during the evaluation phase was rated with the aid of a logbook in which all aspects of interest were recorded.

As a result of all logbooks, only a few problems or incidents have to be mentioned here. In one laboratory, the liquid level detection for reagent pipetting appeared to work incorrectly just before a reagent bottle change-over leading to an incorrect result without any flag. This error could not be reproduced during the further study.

The motor of the operation unit stand was defect at one site. As a consequence, the screen could not be adjusted correctly to the height of the operator. The defect was repaired by exchanging the motor. A further laboratory reported an alarm of abnormal ISE syringe movement which was observed only once.

### *Assessment of practicability*

The practicability of the BM/Hitachi 917 was judged in comparison with the present situation in the evaluating laboratories. The median of all laboratories was calculated from the mean of all scores obtained from each group of attributes. These results are shown in figure 7. More detailed information on the distribution of scores in relation to the main topics is given in figure 8.

### Discussion

The new selective access analysis system BM/Hitachi 917 was evaluated in an international multicentre study, mainly according to the ECCLS criteria for the evaluation of analysers in clinical chemistry. Forty-three different analytes, covering 56 different methods—enzymes, substrates, electrolytes, specific proteins, drugs and urine applications—were tested in seven European clinical chemistry laboratories. Additionally, the practicability of the BM/Hitachi 917 was tested according to a standardized questionnaire.

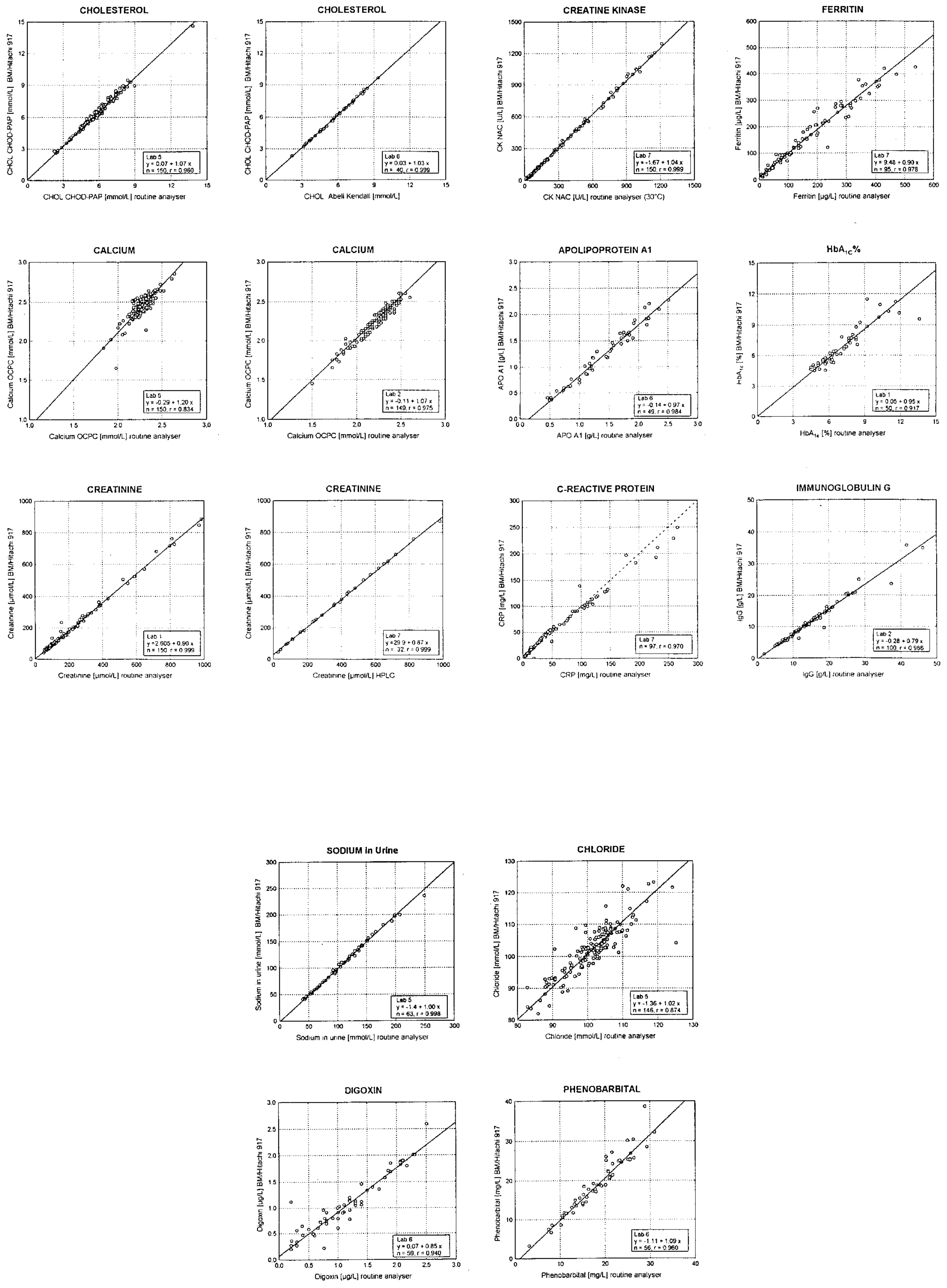

*Figure 6. Method comparison.*

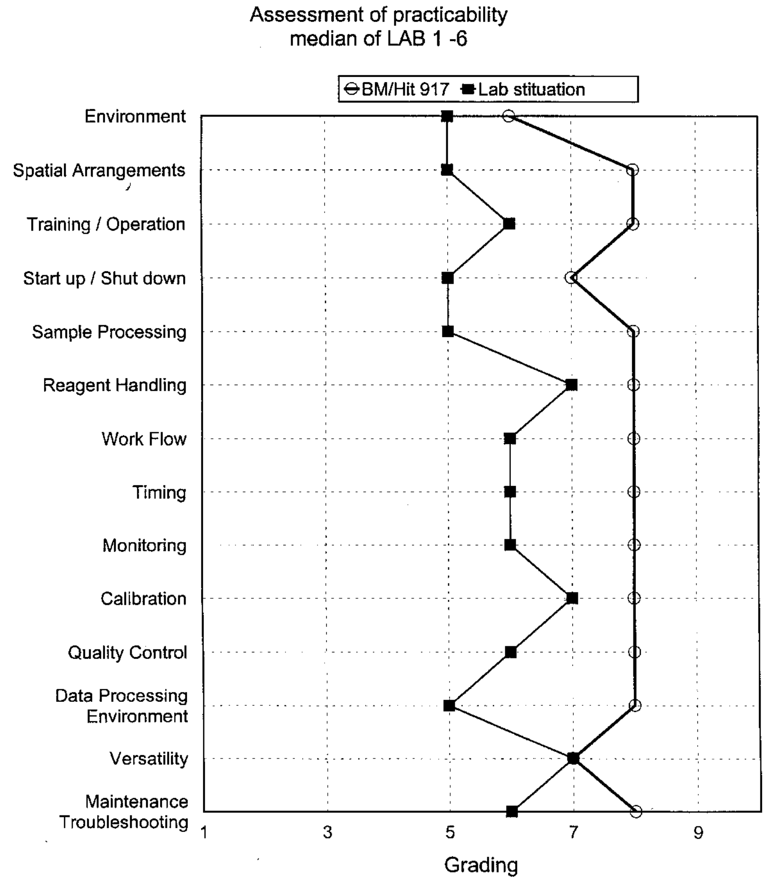

*Figure 7. Assessment of practicability (median of scores).*

A good performance was found for most of the analytes in all laboratory sections using different sample materials of serum, plasma and urine. Although some of the analytes did not fulfil the acceptance criteria, none could be rated as unacceptable. With more than 120 000 individual data, it is impossible to discuss all the results, we therefore selected mainly the results outside the acceptance criteria for discussion.

Regarding the analytical evaluation, and starting with the precision study, we found very satisfying results overall with only a few exceptions (see tables 4 and 5). In the enzyme, substrate and electrolyte section, we only found two outlying results with the human pool sample in measuring the within-run imprecision, maybe because of the (low) concentration of creatine-kinase MB and lipase. For proteins, drugs and urine analytes, all within-run median CV results were lower than 4%, except for digoxin (4.5%) and albumin in urine (4.3%)

Concerning the between-day CVs, here too, very few results exceeding the acceptance limits were observed, i.e. lipase, creatine-kinase and MB isoform, D-dimer, glycosylated haemoglobin, rheumatoid factor, digoxin, digitoxin, theophylline and albumin in urine in some materials. In all situations, the comparison methods gave equivalent results with the exception of glycosylated haemoglobin.

Comparing the results of the between-day imprecision measurements with the Fraser criteria based on within-subject biological variation [13, 14], it is justified to say that the BM/Hitachi 917 achieved these criteria for all analytes except sodium, chloride, digitoxin and phenobarbital. For sodium and chloride it should be stated that the biological varition is that low that no available technology of today can fulfil these criteria.

The linearity, drift and carry-over study showed results all satisfying the test specifications. One exception is the carry-over effect of 5.3 mg/l albumin in urine, which is beyond the acceptance limits of 1.0 mg/l specified by the manufacturer. Measurements performed on two instruments at Boehringer Mannheim resulted in a carry-over effect of 1.7 mg/l, which still requires the use of a evasion procedure.

The interference results are given in table 8, clearly showing that also for the BM/Hitachi 917 interference exists sometimes, as could be expected because of the chemistries applied. It is a situation that can be found in equivalent analysers as well [2].

The method comparisons were often performed with other Hitachi instruments except for the exoteric tests. Most of the 160 comparisons showed acceptable agreement. Nevertheless, some deviations were found, also caused by discrepancies in the evaluation group methods. Striking examples in this respect are ASAT, ALAT, chloride, sodium and HbA1c (results not shown). The question arises whether the tolerance limits (see Introduction) are applicable here.

Further, we want to point out some exceptional results. It was, e.g. remarkable that creatinine deviated in the

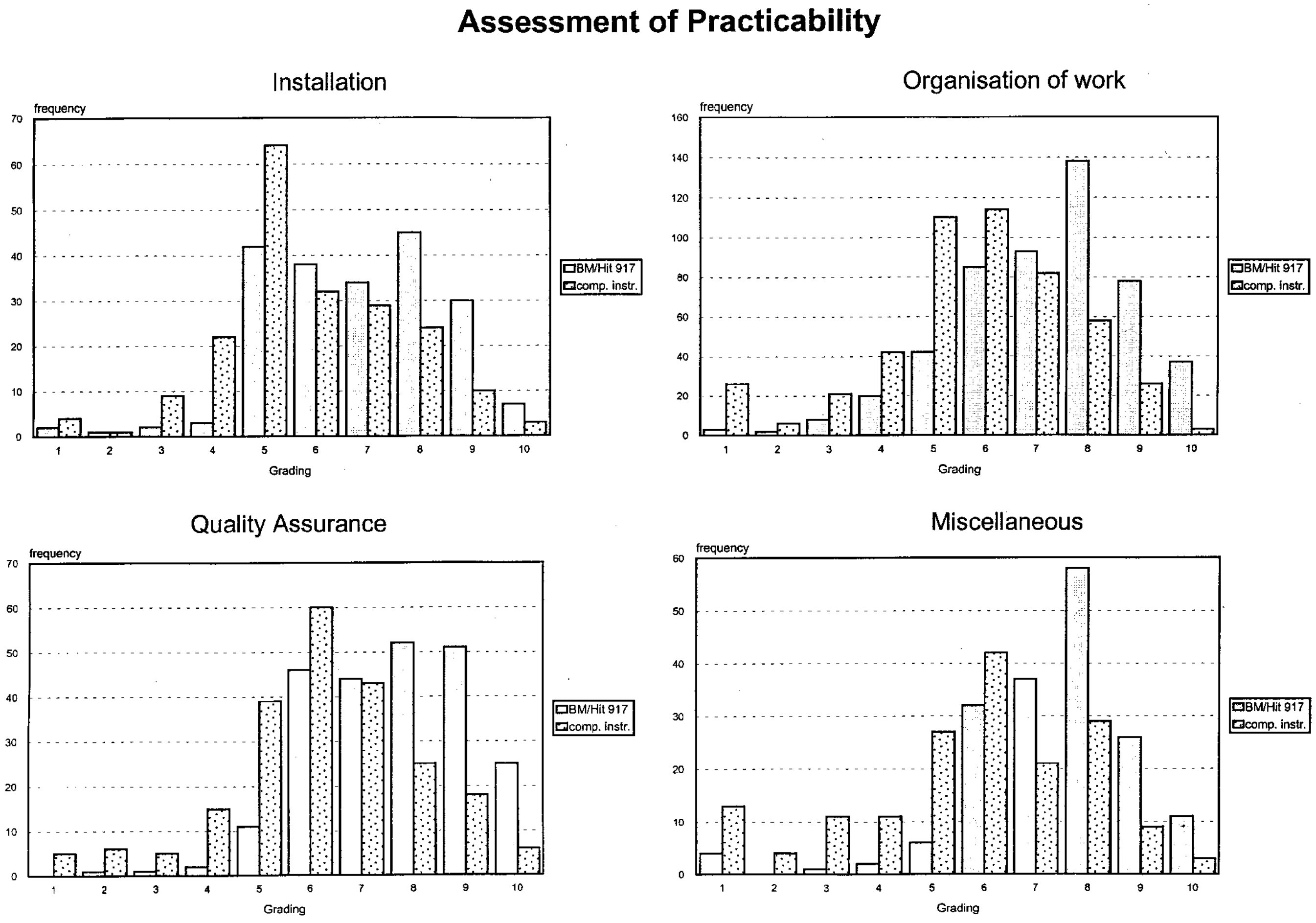

*Figure 8. Assessment of practicability (distribution of scores of the main attribute groups).*

comparison with the HPLC reference method (see figure 6). This deviation was also found in the NIST materials. According to the information of the manufacturer, this problem is under study now. C-reactive protein also showed a remarkable picture: results lower than 100 mg/l showed a different regression equation from results higher than 100 mg/l (see figure 6). We have no explanation for this phenomenon. The regression equations and figures of all additional analytes are available on request.

In a multicentre evaluation, usually the main interest is the evaluation of the analytical performance. Additional to that performance, we thoroughly tested reliability and practicability of the BM/Hitachi 917 as well. Particulary in those stages of the evaluation, the analyser appeared to be a multi-purpose analyser with benefits exceeding those of comparable analysis systems. As can be seen from figure 7, the assessment of practicability for 14 groups of attributes resulted in a grading of one–three scores better for BM/Hitachi 917 than the present laboratory situation.

The system offers features making it attractive to different purposes and different sections in clinical chemistry. It can either be used as a high-throughput analyser for the basic clinical chemistry tests, or as a consolidated workstation for the determination of at least 48 different analytes (on board), covering besides the classical routine assays, specific protein methods, drug methods in serum and urine, and urinalysis applications for enzymes, substrates, electrolytes and proteins.

In the opinion of the authors, laboratory consolidation in combination with laboratory automation is the future of clinical chemistry. The BM/Hitachi 917 therefore can not only be seen as a valuable analyser for the laboratories of today, but also fits in the organizational structures of the future.

### Notes

(1) Most of the practical work was performed during 1996, part of it in early 1997.

(2) Despite the 1998 takeover of Boehringer Mannheim by Roche, we used the term BM/Hitachi 917 because of its wide international acceptance.

### Acknowledgment

The authors would like to thank all technical personnel at the various locations for their valuable and dedicated support.

*Table 9. Control and standard reference materials.*

| *Short name* | *Description* | *Lot. no.* | *Experiments* |
|---|---|---|---|
| CAL | Calibrator routine | | BI |
| CFAS | Cfas, H917 calibrator | 187184-01 | BI, D |
| CFAS-PROT | Cfas Protein, H917 calibrator | 187044-01 | BI |
| COMP | Compensator for H917/ISE | 186654-61 | BI |
| COMP-R | Compensator for Routine | | BI |
| PM RF | Precimat RF | 653015-01 | |
| PNU | Control Serum PNU | 182658-01 | WI, BI, D |
| PPU | Control Serum PPU | 186269-02 | WI, BI, D, CO-C/P |
| PN CK-MB | Precinorm CK-MB | 184932-61 | WI, BI, QC |
| PN PROT | Precinorm Protein | 185861-03 | WI, BI, QC |
| PP PROT | Precipath Protein | 186653-01 | WI, BI, QC |
| PN L | Precinorm Lipid | 185597-01 | WI, BI, QC |
| PP L | Pricipath Lipid | 183253-64 | WI, BI, QC |
| PN RF | Precinorm RF | 654570-01 | WI, BI, QC |
| PN S | Precinorm S | 174973-72 | WI, BI, QC |
| PP S | Precipath S | 181413-64 | WI, BI, QC |
| PN HBA1C | Precinorm HBA1C | 187821-01 | WI, BI, QC |
| PP HBA1C | Precipath HBA1C | 187820-01 | WI, BI |
| C1 D-DIMER | Control 1 D-Dimer | 655266-01 | WI, BI |
| C2 D-DIMER | Control 2 D-Dimer | 655266-01 | WI, BI |
| TDM II | PN TDM, level II | 186618-01 | WI, BI, QC |
| TDM III | PN TDM, level II | 186618-01 | WI, BI |
| SRM | Ring Trial sample for substrates and electrolytes | 909A | QA |
| QC PNU | Control Serum PNU | 185229-08 | QC |
| QC PPU | Control Serum PPU | 179000-05 | QC |
| QC RT | QC Ring Trial | 187746 Cfas | QA |
| CRM 426 | Ring Trial sample for ALAT | | QA |
| CRM 371 | Ring Trial sample for ALP | | QA |
| CRM 299 | Ring Trial sample for CK | | QA |
| CRM 319 | Ring Trial sample for GGT | | QA |
| HPP | Human plasma pool | | WI, BI, Lin |
| HPP-SAT | Human plasma pool for satellite analytes | | WI, BI |
| HSP | Human serum pool | | WI, BI, Lin |
| HEMOLYSATE | Human hemolysed blood | | WI, BI, Lin |
| HSP-CKMB | Human serum pool with CK-MB | | WI, BI, Lin |
| HSP-DIGOX | Human serum pool with Digoxin | | WI, BI, Lin |
| HSP-PHEBA | Human serum pool with Phenobarbital | | WI, BI, Lin |
| HSP-SAT | Human serum pool for satellite experiments | | WI, BI, Lin |
| HSP-THEO | Human serum pool with Theophylline | | WI, BI |
| HP | Human plasma | | MC, I, CO-S |
| HS | Human serum | | MC, I, CO-S |
| HS-CKMB | Human serum with CK-MB | | MC, I |
| HS-DIGIT | Human serum with digitoxin | | MC, I |
| HS-DIGOX | Human serum with digoxin | | MC, I |
| HS, PHEBA | Human serum with Phenobarbital | | MC, I |
| HS-THEO | Human serum with Theophylline | | MC, I |
| CT A1 | Control alpha 1 Microglobulin | Eval. Lot. | WI, BI |
| PN A1 | Precinorm alpha 1 Microglobulin | Eval. Lot. | WI, BI |
| LYPHO1 | Lyphocheck 1 | 62021 | WI, BI, QC |
| LYPHO2 | Lyphocheck 2 | 62022 | WI, BI, QC |
| PN ALB | Precinorm Albumin | 186312-01 | WI, BI, QC |
| PP ALB | Precipath Albumin | 187289-01 | WI, BI |
| HUP | Human urine pool | | WI, BI |
| HU | Human urine | | MC, CO-S |

### References

1. Stockmann, W., Bablok, W., Hollmann, A. and McGovern, M., 1995, Analytical performance of the Boehringer Mannheim/ Hitachi 917 analysis system. *Quimica Clinica*, **14**(S), 294.
2. Zaman, Z., Blankaert, N., Cobbaert, Ch., Gillery, P., Hagemann, P., Luthe, H., Motta, R., Patrono, D., Jionsué, M.-A., Torralba, A., Castiñeras, M. J., Fuentes-Arderiu, X., Bablock, W., Domke, I. and Stockmann, W., 1993, Multicentre evaluation of the Boehringer Mannheim/Hitachi 911 Analysis System. *Journal of Automatic Chemistry*, **15**, 189–208.
3. Haeckel, R., Busch, E. W., Jennings, R. D. and Truchaud, A., 1986, Guidelines for the evaluation of analysers in clinical chemistry. ECCLS, document 3: No. 2, Beuth, Berlin.
4. Bablok, W., Barembruch, R., Stockmann, W., Brauer, P., Graber, P., Michel, R. and Vonderschmitt, D. J., 1991, CAEv—A program for computer aided evaluation. *Journal of Automatic Chemistry*, **13**, 167–179.

5. Hafner, G., Endler, Th., Oppitz, M., Merten, U. P., Töpfer, G., Dubois, H., Hallstein, A., Hilger, B. and Domke, I., 1995, Effects of standardization with the new international reference preparation for proteins in human serum on method comparability and reference values. *Clin. Lab.*, **41**, 743–748.
6. Bablok, W., 1993, Range of linearity. In R. Haeckel (ed.) *Evaluation Methods in Laboratory Medicine* (Weinheim, Germany: VCH), pp. 251–258.
7. Passing, H. and Bablok, W., 1983, A new biometrical procedure for testing the equality of measurements from two different analytical methods. *J. Clin. Chem. Clin. Biochem.*, **21**, 709–720.
8. Broughton, P. M. G., Gowenlock, A. H., McCormack, J. J. and Neill, D. W., 1974, A revised scheme for the evaluation of automatic instruments for use in clinical chemistry. *Ann. Clin. Biochem.*, **11**, 207.
9. Glick, M. R., Ryder, K. W. and Jackson, S. A., 1986, Graphical comparisons of interferences in clinical chemistry instrumentation. *Clin. Chem.*, **32**, 470–475.
10. Bablok, W. and Stockmann, W., 1995, An alternative approach to a system evaluation in the field. *Quimica Clinica*, **14**(S), 239.
11. Stockmann, W., Bablok, W., Poppe, W., Bayer, P. M., Keller, F. and Schweiger, C. R., 1993, Criteria of practicability. In H. Haeckel (ed.) *Evaluation Methods in Laboratory Medicine* (Weinheim, Germany: VCH), pp. 185–201.
12. Bonini, P., Ceriotti, F., Keller, F., Brauer, P., Stolz, H., Pascual, C., Beltrán, L. G., Vonderschmitt, D. J., Pei, P., Bablok, W., Domke, I. and Stockmann, W., 1992, Multicentre evaluation of the Boehringer Mannheim/Hitachi 747 analysis system. *Eur. J. Clin. Chem. Clin. Biochem.*, **30**, 881–899.
13. Fraser, C. G., Hyltoft Petersen, P., Ricos, C. and Haeckel, R., 1993, Criteria for imprecision. In H. Haeckel (ed.) *Evaluation Methods in Laboratory Medicine* (VCH), p. 87.
14. Fraser, C. G., Hyltoft Petersen, P. H.., Ricos, C. and Haeckel, R., 1992, Proposed quality specifications for the imprecision and inaccuracy of analytical systems for clinical chemistry. *Eur. J. Clin. Chem. Clin. Biochem.*, **30**, 311–317.

